# 

**Published:** 1908-02-22

**Authors:** 


### BOOKS RECEIVED.

J. and A. Churchill.

"A Short Practice of Gynæcology." 3rd edition. By H. Jellett, B.A., M.D.

"A Manual of Prescribing." By C. R. Marshall, M.D.

Hazell, Watson, and Viney, Ltd.

"Hazell's Annual, 1908."

Marshall Bros., Ltd.

"Christian Sanity." By A. T. Schofield, M.D.

John Murray.

"The Licensed Trade." By Edwin A. Pratt.

Hy. Frowde and Hodder and Stoughton.

"Life Insurance and General Practice." By E. M. Brockbank, M.D.

"Rotunda Medical Midwifery" By E. Hastings Tweedy, M.D., and G. T. Wrench, M.D.

"Fevers in the Tropics." By Leonard Rogers, M.D.

The Keystone Press, Philadelphia.

"The Ocular Muscles." By Ernest E. Maddox, M.D.

Their Majesties the King and Queen have announced their intention of being present at an invitation concert to be given at the Albert Hall in May next to Vice-Presidents and members who have worked for the League of Mercy for two years and upwards. Many leading artistes have kindly offered their services. There will be a limited number of tickets for sale. Further particulars will be given in due course.

### THE GRADUATES' MEDICAL SCHOOLS.

### Diary for the week February 24 to 29.

**THE ROYAL COLLEGE OF SURGEONS.**

**At 5 p.m.**
**Feb. 24, 26, and 28,** Prof. Donald Armour, **The Surgery of the Spinal Cord nnd its Membranes.**

**THE HOSPITAL FOR SICK CHILDREN, Great Ormond Street, W.C.**

**At 4 p.m.**
**Feb. 27,** Mr. Fairbank, **Surgical Treatment of Paralysis.**

**HOSPITAL FOR CONSUMPTION AND DISEASES OF THE CHEST, Brompton, S.W.**

**At 4 p.m.**
**Feb. 26,** Dr. Fenton, **The Myogenic Theory in Relation to Cardiac Arrhythmia.**

**LONDON SCHOOL OF CLINICAL MEDICINE, Seamen's Hospital, Greenwich.**

**At 3.15 p.m.**
**Feb. 25,** Mr. Carless.
**At 2.15 p.m.**
**Feb. 28,** Dr. Rose Bradford, **Diabetes.**

**MOUNT VERNON HOSPITAL, Fitzroy Square.**
Post-Graduate Course.

**At 5 p.m.**
**Feb. 27,** Dr. F. W. Price, **The Early Diagnosis of Pulmonary Tuberculosis.**

**CHARING CROSS HOSPITAL, W.C.**
Post-Graduate Course.

**At 4 p.m.**
**Feb. 27,** Dr. Fenton, **Medical.**

**NATIONAL HOSPITAL FOR THE PARALYSED AND EPILEPTIC, Queen Square, Bloomsbury, W.C.**

**At 3.30 p.m.**
**Feb. 25,** Dr. R. Russell, **Cerebellar Disease.**
**Feb. 28,** Sir V. Horsley, F.R.S., **Surgery of the Nervous System.**

**THE POST-GRADUATE COLLEGE, West London Hospital, Hammersmith, W.**

**At noon.**
**Feb. 24,** Dr. Low, **Pathological Demonstration.**
**Feb. 25 and 27,** Dr. Pritchard, **Practical Medicine.**
**At 5 p.m.**
**Feb. 24,** Dr. Kenneth Scott, **Some Acute Inflammations of the Eye.**
**Feb. 25,** Dr. Low, **Plague.**
**Feb. 26,** Dr. Beddard, **Practical Medicine—IV.**
**Feb. 27,** Mr. Keetley, **Clinical Lecture—II.**
**Feb. 28,** Dr. Seymour Taylor, (*a*) **The Significance of Urates;** (*b*) **The Significance of a Trace of Albumen.**

**THE HOSPITAL FOR NERVOUS DISEASES, Welbeck Street, W.**

**At 5 p.m.**
**Feb. 27,** Dr. F. S. Palmer, **Tabes Dorsalis.**

**NORTH-EAST LONDON POST-GRADUATE COLLEGE, Prince of Wales' General Hospital, Tottenham, N.**

**At 4.30 p.m.**

**Feb. 27,** Dr. A. G. Auld, **Asthma, its Pathology and Treatment.**

**THE THROAT HOSPITAL, Golden Square, W.**

**At 5.30 p.m.**

**Feb. 24,** Mr. C. A. Parker, **Chronic Inflammatory Affections—Ætiology and Symptoms.**

**Feb. 27,** Mr. C. A. Parker, **Chronic Inflammatory Affections—Treatment.**

**MEDICAL GRADUATES' COLLEGE AND POLYCLINIC, Chenies Street, W.C.**

**Lectures at 5.15 p.m.**

**Feb. 24,** Mr. B. G. A. Moynihan, **The Differential Diagnosis of Obstructive Jaundice.**

**Feb. 25,** Dr. Lewis Smith, **Syphilis in General Medicine.**

**Feb. 26,** Dr. Leslie Paton, **The Retina in Albuminuria.**

**Feb. 27,** Dr. Frederick Taylor, **Leukæmia.**

THE HOSPITAL *February* 22, 1908.
*Name* ..........................................
*Address* ..........................................
..........................................
This Coupon must accompany manuscript or contributions intended for THE HOSPITAL.

